# Infection-related hospitalization following ureteroscopic stone treatment: results from a surgical collaborative

**DOI:** 10.1186/s12894-020-00720-4

**Published:** 2020-11-03

**Authors:** Adam Cole, Jaya Telang, Tae-Kyung Kim, Kavya Swarna, Ji Qi, Casey Dauw, Brian Seifman, Mazen Abdelhady, William Roberts, John Hollingsworth, Khurshid R. Ghani, for the Michigan Urological Surgery Improvement Collaborative

**Affiliations:** 1 Department of Urology, University of Michigan, Ann Arbor, MI 48103 USA; 2 Michigan Institute of Urology, West Bloomfield, MI 48322 USA; 3 Detroit Medical Center, Department of Urology, Detroit, MI 48201 USA

**Keywords:** Ureteroscopy, Infection, Outcomes, Quality improvement, Urolithiasis

## Abstract

**Background:**

Unplanned hospitalization following ureteroscopy (URS) for urinary stone disease is associated with patient morbidity and increased healthcare costs. To this effect, AUA guidelines recommend at least a urinalysis in patients prior to URS. We examined risk factors for infection-related hospitalization following URS for urinary stones in a surgical collaborative.

**Methods:**

Reducing Operative Complications from Kidney Stones (ROCKS) is a quality improvement (QI) initiative from the Michigan Urological Surgery Improvement Collaborative (MUSIC) consisting of academic and community practices in the State of Michigan. Trained abstractors prospectively record standardized data elements from the health record in a web-based registry including patient characteristics, surgical details and complications. Using the ROCKS registry, we identified all patients undergoing primary URS for urinary stones between June 2016 and October 2017, and determined the proportion hospitalized within 30 days with an infection-related complication. These patients underwent chart review to obtain clinical data related to the hospitalization. Multivariable logistic regression analysis was performed to determine risk factors for hospitalization.

**Results:**

1817 URS procedures from 11 practices were analyzed. 43 (2.4%) patients were hospitalized with an infection-related complication, and the mortality rate was 0.2%. Median time to admission and length of stay was 4 and 3 days, respectively. Nine (20.9%) patients did not have a pre-procedure urinalysis or urine culture, which was not different in the non-hospitalized cohort (20.5%). In hospitalized patients, pathogens included gram-negative (61.5%), gram-positive (19.2%), yeast (15.4%), and mixed (3.8%) organisms. Significant factors associated with infection-related hospitalization included higher Charlson comorbidity index, history of recurrent UTI, stone size, intra-operative complication, and procedures where fragments were left in-situ.

**Conclusions:**

One in 40 patients are hospitalized with an infection-related complication following URS. Awareness of risk factors may allow for individualized counselling and management to reduce these events. Approximately 20% of patients did not have a pre-operative urine analysis or culture, and these findings demonstrate the need for further study to improve urine testing and compliance

## Background

Ureteroscopy (URS) is now the most common treatment modality for treating upper urinary tract stones in North America [1, 2]. Due to technological advances and widespread availability of equipment, URS is often performed in the outpatient setting [3]. Despite this, morbidity, especially infection-related complications, may occur in up to 5–18% of patients [4–8]. These often result in hospital admission and can have a significant impact on patients, providers, and payers [3, 9–11]. A hospital admission for sepsis can cost approximately $20,000 [12]. Therefore, efforts to mitigate infection-related complications following URS would be beneficial in reducing healthcare expenditures.

Prior studies investigating infection-related complications after URS have provided some insights on risk factors, which include stone, patient, and operative characteristics. However, most are single institution series from tertiary referral or academic medical centers [4–10], which may limit generalizability of the results to the wider swathe of urologic patients commonly treated by diverse practitioners in community or multi-specialty group practices.

In the state of Michigan, we have developed a quality improvement (QI) initiative and a clinical registry—Reducing Operative Complications from Kidney Stones (ROCKS)—to better understand processes of care, outcomes, and quality indicators for patients undergoing URS for urinary stones. A strength of this registry is its diversity of patients and practicing urologists. In our drive to improve outcomes for URS, we sought to better understand risk factors for infection-related hospitalization using data from this surgical collaborative. We also sought to assess care in relation to guideline-based practice. We hypothesize that there are modifiable factors which lead to infection related morbidity. Identifying high-risk patients may allow for individualized counseling, and development of QI interventions that reduce adverse events, and the associated patient morbidity and healthcare costs.

## Methods

### Data source

The Michigan Urological Surgery Improvement Collaborative (MUSIC) was established in 2011 in partnership with Blue Cross Blue Shield of Michigan. The ROCKS QI initiative within MUSIC comprises diverse community and academic urology practices in the state of Michigan and started in 2016. For patients with urinary stones undergoing URS, trained abstractors prospectively record standardized data elements in a web-based registry including patient and stone characteristics, surgical details and complications. Patient data are entered into the registry 60 days after a URS procedure, and data entry is guided by standard variable definitions and collaborative-wide operating procedures. To ensure data quality, the coordinating center performs on-site data audits on a semi-annual basis.

### Patient selection and outcomes

We identified all patients undergoing URS for primary treatment of urinary stones between June 2016 and October 2017. During this period, ROCKS consisted of 11 practices. To be included in the ROCKS registry, a patient had to be at least 18 years of age and undergone unilateral URS for urinary stones. Patients who underwent bilateral URS, had an ipsilateral nephrostomy at the time of URS, or underwent URS after percutaneous renal surgery were ineligible. We identified all patients who were discharged after surgery and then subsequently hospitalized (at any institution) within 30 days of their procedure. An infection-related hospitalization was determined by chart review, based on the presence of SIRS criteria with or without bacteriuria. Patients admitted for other indications (pain, hematuria, etc.) were classified as a non-infectious hospitalization. Stone-free rate (SFR) was defined as absence of any fragment on X-ray, CT or ultrasound reports obtained within 60 days. Chart review was performed on all patients with infection-related hospitalizations, including urine culture pathogen data, length of stay, and timing from surgery.

### Statistical analyses

We generated descriptive summary statistics of all patients in the analytic sample. Chi-square tests and student’s *t* tests were performed for categorical and continuous variables, respectively, to compare demographic and operative factors between the two groups. Significant pre-operative and operative variables were then used as covariates in a multivariable analysis to determine which factors were associated with higher odds of an infection-related hospitalization. Multivariable analysis was performed using a logistic regression model. The odds ratios and 95% confidence intervals were reported. Significant variables with less than 10 events were not included in the multivariable final model. All analyses were performed with SAS 9.4 (SAS Institute, Cary, NC) at a 5% significance level.

## Results

**Fig. 1:**
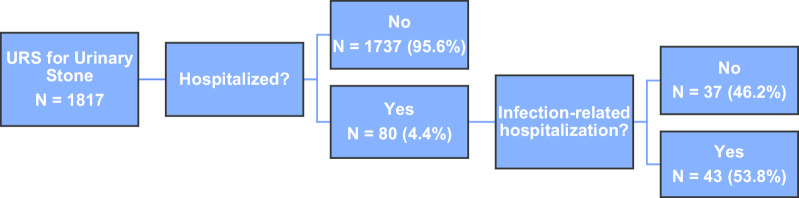
Post-operative hospitalization events following ureteroscopy for urinary stones in 1817 patients in a statewide surgical collaborative (MUSIC)

A total of 1817 URS procedures in 1737 patients from 11 practices were analyzed. In total, 80 (4.4%) patients were hospitalized within 30 days of their URS. Forty-three (2.4%) patients were hospitalized with an infection-related complication (Fig. 1). Median time from surgery to admission was 4 days (range 0–30) and median length of stay was 3 days (range 1–33) for the patients admitted with an infection-related complication. The majority of admissions (74.4%) occurred within 7 days of surgery, and more than half of patients (55.8%) were admitted for longer than 2 days. One (2.3%) patient had a prior ureteroscopy within 1 month of the index surgery. Of patients with a positive urine culture during hospitalization (n = 26), isolated pathogens included 16 (61.5%) gram-negative, 5 (19.2%) gram-positive, 4 (15.4%) yeast, and 1 (3.8%) with gram-positive and -negative cocci. Only 9 of these 26 patients (34.6%) had positive urinalysis (defined by positive nitrite) or urine culture prior to surgery. Three patients died during their hospitalization (mortality rate 0.2%).

Pre-operative, intra-operative, and post-operative characteristics among infection-related hospitalized, and non-hospitalized patients, are provided in Table 1. Significant factors for hospitalization with an infection-related complication on bivariate analysis were public insurance status, older age, higher Charlson Comorbidity Index (CCI), history of recurrent UTI (registry variable based on clinic note by physician indicating history of prior UTIs), spinal cord injury, urinary diversion, intra-operative complication, and larger stone size. Patients that were hospitalized were less likely to be on pre-operative alpha-blockers. There was no statistical difference in the proportion of patients who had an indwelling ureteral stent prior to URS. Of those hospitalized with an infection-related complication, 9 (20.9%) did not have a pre-procedure urinalysis or urine culture, compared to 355 (20.5%) in the non-hospitalized group (*p* = 0.95). 12 (27.9%) patients who were hospitalized with an infectious indication had a positive urinalysis or urine culture prior to surgery, compared to 261 (15.4%) in the non-hospitalized patients (*p* = 0.08). None of the 12 patients with abnormal pre-operative urine studies who were hospitalized were treated with antibiotics prior to surgery.

**Table 1:** Patient characteristics for patients undergoing ureteroscopy for urinary stones in MUSIC ROCKS stratified by post-operative course

Risk factor	Infection-related hospitalization (n = 43)	Non-hospitalized (n = 1737)	*P *value
*Pre-operative characteristics*			
Public insurance	25 (58.1%)	669 (39.7%)	0.01
Mean age (SD)	60.1 (15.8)	54.4 (15.5)	0.02
Male gender	19 (44.2%)	867 (50.1%)	0.44
BMI > 30	24 (58.5%)	788 (46.8%)	0.13
CCI ≥ 1	26 (60.5%)	495 (28.5%)	< 0.01
CCI ≥ 2	14 (32.6%)	241 (13.9%)	< 0.01
Presence of hydronephrosis on pre-operative imaging	27 (67.5%)	1048 (66.7%)	0.92
Largest stone size (mm), mean (SD)	10.1 (6.5)	7.8 (5.4)	< 0.01
Solitary kidney	2 (4.7%)	25 (1.5%)	0.13
Horseshoe kidney	1 (2.3%)	6 (0.4%)	0.16
History of recurrent UTI	9 (20.9%)	88 (5.1%)	< 0.01
Urinary diversion	2 (4.7%)	7 (0.4%)	0.02
Spinal cord injury	2 (4.7%)	3 (0.2%)	< 0.01
Anti-platelet therapy	13 (30.2%)	345 (20.4%)	0.12
Pre-operative urinalysis/urine culture not performed	9 (20.9%)	355 (20.5%)	0.95
Positive pre-operative urinalysis/urine culture	12 (27.9%)	266 (15.4%)	0.08
Positive pre-operative urinalysis/urine culture treated	0 (0%)	59 (22.2%)	0.07
Urgent/emergent surgery	1 (2.3%)	146 (8.4%)	0.15
Peri-operative antibiotic use	38 (95%)	1513 (96.9%)	0.49
Alpha-blocker therapy prior to URS	11 (26.2%)	755 (45.1%)	0.01
Pre-stenting (ureteral stent in place)	20 (46.5%)	649 (37.5%)	0.23
*Stone location*			
Renal	17 (45.9%)	502 (31.0%)	0.07
Ureter	14 (37.8%)	906 (56.0%)	
Both	6 (16.2%)	211 (13.0%)	
*Intra-operative characteristics*			
Intra-operative complication	3 (7.0%)	33 (1.9%)	< 0.01
Complication: bleeding	2 (4.7%)	14 (0.8)	
Complication: perforation	0 (0.0%)	4 (0.2%)	
Complication: other	1 (2.3%)	15 (0.9%)	
Ureteral dilation	6 (13.9%)	340 (19.7%)	0.44
Ureteral access sheath use	18 (41.9%)	626 (36.6%)	0.49
Lithotripsy with fragments left in-situ	30 (69.8%)	716 (42.3%)	< 0.01
Stenting during URS	31 (72.1%)	1248 (72.1%)	0.99
*Post-operative characteristics*			
Discharged with antibiotics	15 (36.6%)	638 (39.2%)	0.74
Discharged with antibiotics and stent placed	9 (20.9%)	509 (29.3%)	0.23
Discharged with alpha-blocker	27 (65.8%)	911 (55.9%)	0.21
Stone free rate	19 (57.6%)	579 (77.5%)	< 0.01

Patients who were hospitalized for infectious reasons were more likely to have an intra-operative complication (7.0%). Complications included inability to complete procedure due to bleeding or perforation. There was no difference in the rate of ureteral stent placement, ureteral dilation, or use of ureteral access sheath at the time of surgery between the infection-related hospitalization group and the non-admitted group. Those hospitalized with an infection-related complication were more likely to have lithotripsy with fragments left in situ at the conclusion of the operation.

On multivariable analysis (Table 2), significant risks factors associated with hospitalization for infection-related causes included higher CCI, history of recurrent UTI, increasing stone size, history of intra-operative complication, and lithotripsy with fragments left in-situ. The strongest risk factors were the presence of an intra-operative complication (OR 3.7) and history of recurrent UTI (OR 3.74).

**Table 2:** Multi-variable logistic regression demonstrating association between patient characteristics and risk of infection-related hospitalization

Risk factor	OR	CI	*P *value
Age	1.01	0.98–1.03	0.95
Comorbidity (CCI 0 vs. 1)	3.12	1.37–7.14	< 0.01
Comorbidity (CCI 0 vs. 2)	2.72	1.16–6.37	0.02
Stone size	1.04	1.01–1.07	0.02
History of recurrent UTI	3.74	1.55–9.00	< 0.01
Insurance (public vs. private)	1.57	0.75–3.25	0.23
Alpha-blocker prior to URS	0.51	0.24–1.06	0.07
Complete fragment removal	0.32	0.16–0.65	< 0.01
Intra-operative complication	3.70	1.22–11.25	0.02

## Discussion

We found that in 11 diverse urology practices across the state of Michigan, 1 in 40 patients were hospitalized with an infection-related complication following URS for urinary stones. During admission the most commonly identified organisms were gram-negative, however a small proportion of patients had yeast identified. Risk factors for an infection-related admission were higher Charlson comorbidity index, history of recurrent UTI, larger stone size, intra-operative complication and cases where lithotripsy was performed with fragments left in-situ. Overall, 20% of all patients did not have a documented urinalysis or urine culture prior to URS. Collectively, these findings represent an opportunity for the development of QI initiatives to decrease the risk of infection and sepsis after URS, as well as better adherence to American Urological Association (AUA) guidelines.

Previous investigators have examined risk factors for infectious complications following URS. Zhong et al. examined 250 patients that underwent URS for stone treatment, and found an 8.1% incidence of systemic inflammatory response syndrome (SIRS) following the procedure. Risk-factors included stone size, smaller caliber ureteral access sheath, higher irrigation flow rate, and presence of struvite calculi [8]. Other studies have also identified female gender [5, 6, 13], history of obstructive pyelonephritis [5, 6], positive pre-operative urine culture [5, 6], and prolonged ureteral stent dwell time [5] as risk factors for SIRS/sepsis, with rates of SIRS/sepsis from 0.30–8% [5–8, 14]. We also found similar risk factors for hospitalization related to infectious complications, including higher Charlson comorbidity index, history of recurrent UTI, intra-operative complication, and stone size. Interestingly, female gender and pre-operative ureteral stenting were not risk factors in this analysis. Female gender has been a reported risk factor in some series [5, 6, 13], however in other studies this was not a risk factor [7, 14] suggesting differences in study design. Perhaps a prospective study would be helpful. Additionally, public insurance was associated with an increased risk of an infectious-hospitalization on univariate analysis. However, this association was not seen in our multi-variable model, suggesting that the association of insurance and infection may be due to other factors.

Awareness of risk factors can allow for an individualized approach to pre-operative antibiotic selection, adoption of intra-operative technical factors such as considering a ureteral access sheath or limiting the irrigation flow rate, and post-operative antibiotic therapy in patients at risk for developing sepsis. Since there was a strong relationship between an intra-operative complication and subsequent hospitalization, patients who suffer this event could be considered for prolonged observation in the recovery room or even admission and observation. Likewise, patients with a history of recurrent UTI should be considered for pre-operative urine culture (not urinalysis) and be managed with culture-directed pre-operative antibiotics. While almost all patients received peri-operative antibiotics, more patients in the hospitalized group had an abnormal urine study prior to surgery, and none of these patients were treated with antibiotics. There are a very small number of patients in both groups with untreated positive urine cultures prior to ureteroscopy. This represents a focus for subsequent quality improvement initiatives with the goal to improve pre-operative testing and follow-up.

We found that in patients with a positive urine culture during hospitalization, only 34.6% had a positive UA or urine culture prior to surgery. It is possible this discordance lies in our definition of a positive urinalysis (nitrite positivity), which can be altered by medications such as pyridium. Additionally, any positive pre-op culture, regardless of organism or colony count, is deemed positive. These represent limitations of our study, however, previous studies have also reported discordance between pre-operative, intra-operative and post-operative urine cultures in patients undergoing stone surgery. Paonessa et al. examined pre-operative urine cultures and intraoperative stone cultures in patients undergoing percutaneous nephrolithotomy and found that 9.7% of patients with negative pre-operative urine cultures had positive stone cultures. In patients with both positive pre-operative urine and intra-operative stone cultures, the organisms differed in 13.3%, representing an overall discordance in almost a quarter of cases [15]. Marien et al. also demonstrated 27% discordant voided and upper tract urine cultures after decompression for obstructing stones [16].

The AUA Guidelines on Surgical Management of Stones advises clinicians to obtain a urinalysis prior to URS, and in patients with clinical or laboratory signs of infection, a urine culture should be obtained [17]. EAU Guidelines state a urine culture or urinary microscopy are mandatory before treatment [18]. In our cohort, approximately 20% of patients who were admitted with an infectious complication did not have a urinalysis or urine culture prior to surgery. This aspect of care, where patients are not managed in accordance with current guidelines represents an area for improvement. Interestingly, this rate was similar in the group of non-hospitalized patients. It would appear that obtaining a pre-operative urinalysis or urine culture did not alter the risk of hospitalization for an infection-related reason. One major limitation of our work is that we do not differentiate between urine culture or urinalysis in our registry. Additionally, the pre-operative screening requirements vary by center due to institutional protocols, work-flow, staffing, and resources. Some institutions require a urine culture within 30 days of surgery, while others use urinalysis with reflex culture. Despite these difference, pre-operative urine studies were not obtained in approximately 20% of all patients, likely for a variety of reasons: urine studies may not have been ordered, urine studies were ordered but not performed by the patient, or they were performed at outside institutions but not available. Our findings warrant further investigation to address these quality of care gaps.

Lithotripsy with fragments left in-situ was associated with an increased risk of infection-related hospitalization in our cohort. This variable is determined by review of the operative notes by data abstractors based on key phrases, such as “all fragments were removed,” or “all remaining fragments were 1 mm of less.” The database does not detect specific stone treatment technique, and it is difficult to ascertain if this indicates dusting technique, or a hybrid technique of basketing and dusting. It is possible that our results are confounded by patients with large stone burden. Also, while patients in the non-hospitalized group were more likely to be on pre-operative alpha-blockers, this was not significantly associated with a lower risk of an infection-related hospitalization on multi-variate analysis. In a recent study, 1 week of pre-operative alpha-blocker therapy was associated with lower overall complications after URS [19]. The same mechanism by which alpha-blockers are prescribed to facilitate ureteral stone passage—inhibition of the alpha receptors in the distal ureter and reduced ureteral muscle tone and peristalsis—has been proposed to facilitate ureteroscopy and instrumentation of the ureter [20]. This is an area of interest that will be the subject of future investigation.

Our work has several limitations. First, our patients are located in a single state and it is possible these results are not applicable to all patients in the United States or outside the country. Our registry does not collect information on pre-operative ureteral stent dwell-time, technical intraoperative details such as size of access sheath, irrigation rate, surgical time, and other factors that may place patients at higher risk for developing an infectious complication. In addition, stone cultures are not captured by registry. Additionally, it is possible we are underreporting the number of events based on our study design. From the registry we were able to identify all patients that were hospitalized within 30 days. We then made a determination if the hospitalization was due to infectious or non-infectious etiologies (pain, hematuria, etc.) based on chart review. Along with the admission notes, SIRS criteria were used to determine if the admission was due to infectious-indication, as culture data was not available or obtained after administration of antibiotics. Therefore it is possible that some patients were admitted with infectious-indications without SIRS criteria. Finally, the small number of hospitalization events may alter the fit of our multi-variable model.

Our findings do have several implications. URS is among the most commonly performed urologic surgeries, and unplanned healthcare encounters following URS are not uncommon. We demonstrate suboptimal statewide compliance with guidelines regarding pre-operative urine screening. Efforts should be taken to comply with best practice statements, and this will be the subject of future QI initiatives in MUSIC. In particular we are considering collecting information on which specific pre-operative urine study was performed to determine whether urinalysis is insufficient as a screening tool to mitigate the risk of sepsis after URS.

## Conclusion

We found that nearly 1 in 40 patients are hospitalized with an infection-related complication following URS for urinary stones in diverse practices in Michigan. Awareness of risk factors may allow for individualized counselling and management to reduce these events. Approximately 20% of patients did not have a pre-operative urine analysis or culture, and these findings demonstrate the need for further study to improve urine testing and compliance.

## Data Availability

The datasets generated and/or analyzed during the current study are not publicly available, and are managed by the MUSIC urology coordinating center. MUSIC urology was founded with the guiding principle to improve the urologic care across the entire state. We do not compare institutions within our registry for the purposes of maintaining confidentiality. As such, our data are internally maintained and not publically available.

## Abbreviations

URS: Ureteroscopy
AUA: American Urological Association
ROCKS: Reducing Operative Complications from Kidney Stones
MUSIC: Michigan Urological Surgery Improvement Collaborative
QI: Quality improvement
SFR: Stone-free rate
UTI: Urinary tract infection
CCI: Charlson Comorbidity Index
OR: Odds ratio
SIRS: Systemic inflammatory response syndrome
UA: Urinalysis
EAU: European Association of Urology
